# Subacute Appendicular Abscess Masquerading as Neoplasia Causing Large Bowel Obstruction

**DOI:** 10.14309/crj.0000000000001085

**Published:** 2023-06-28

**Authors:** Yamini Katamreddy, Olaniyi Fadeyi, Thomas J. Konturek

**Affiliations:** 1 Department of Internal Medicine, West Anaheim Medical Center, Anaheim, CA; 2 Department of Gastroenterology, West Anaheim Medical Center, Anaheim, CA

**Keywords:** subacute, chronic, appendicitis, appendicular abscess, large bowel obstruction

## Abstract

Acute appendicitis is the most common reason for emergency abdominal surgery worldwide. Nonacute appendicitis variants include recurrent, subacute, and chronic appendicitis. Although these are not considered surgical emergencies, they are frequently overlooked, resulting in complications such as perforation or abscess formation. The presentation of nonacute forms is rare in the modern era because of sophisticated diagnostic modalities and treatment measures. We discuss a rare case of subacute appendicular abscess simulating a neoplasm with large bowel obstruction.

## INTRODUCTION

Chronic or subacute appendicitis is more uncommon than acute appendicitis. An appendicular abscess occurs between 2% and 10% of patients with appendicitis.

## CASE REPORT

A 23-year-old woman with no medical history presented with fever and abdominal pain for 1 week. She had noticed a change in the caliber of the stool associated with improvement in pain with bowel movements. The patient denied hematochezia or diarrhea. She had similar complaints 4 months ago, which self-resolved. Her vitals were unremarkable, with laboratory tests showing microcytic anemia. The patient also had mild leukocytosis of 12,800/mL and was started on piperacillin and tazobactam for possible intra-abdominal infection. On examination, the abdomen was soft with tenderness over the left lower quadrant, without peritoneal signs. Abdominal and pelvic CT without oral or intravenous contrast shows a 3 × 3-cm soft-tissue mass of undetermined etiology attached to the lateral aspect of the mid-descending colon.

**Figure 1.**
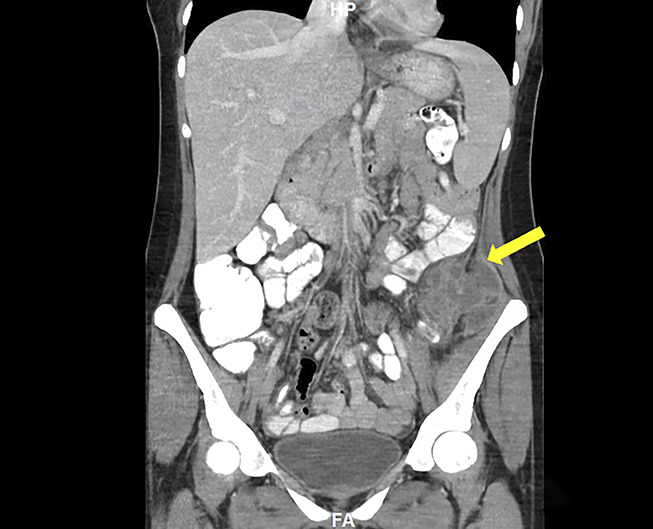
Abdominal and pelvic CT with oral and IV contrast (axial view) revealed mucosal thickening of a short segment of the descending colon measuring 5 cm in length (yellow arrow), causing severe lumen narrowing and partial obstruction of indeterminate etiology. CT, computed tomography; IV, intravenous.

**Figure 2.**
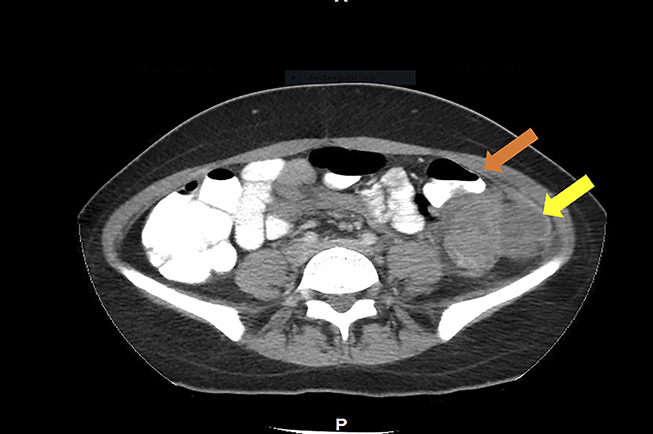
Abdominal and pelvic CT with oral and IV contrast (sagittal view) revealed severe mucosal thickening of a short segment of the descending colon measuring 5 cm in length (yellow arrow), causing severe lumen narrowing and partial obstruction of indeterminate etiology (orange arrow). CT, computed tomography; IV, intravenous.

**Figure 3.**
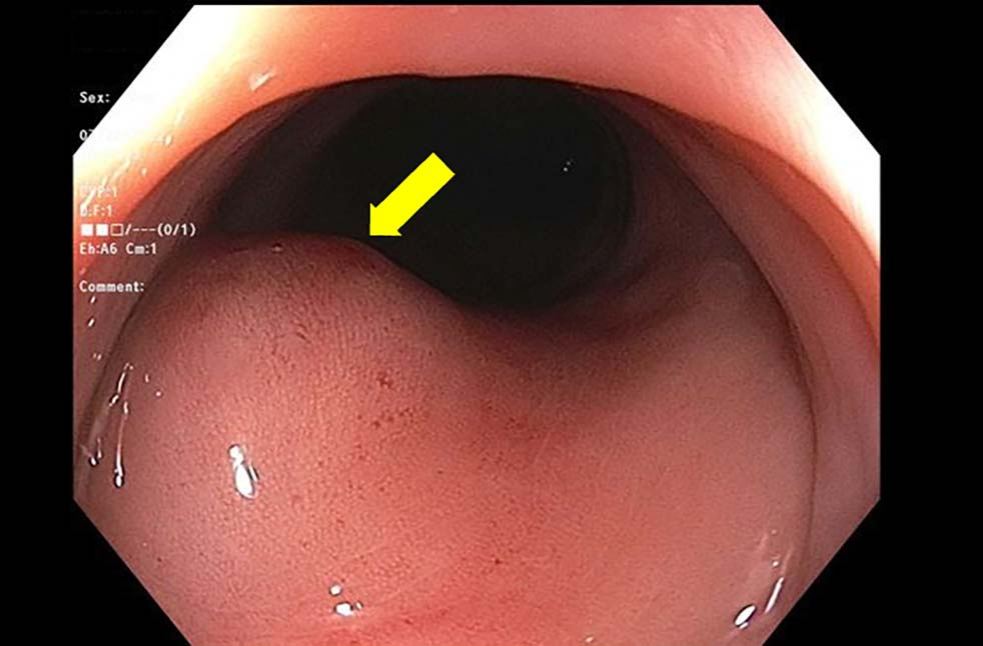
Colonoscopy showed a 4-cm protruding mass (yellow arrow) with compression noticed in the descending colon, with partial obstruction of the lumen corresponding to the area of CT findings. There was no associated colonic inflammation ruling out colitis or inflammatory bowel disease. CT, computed tomography.

Abdominal and pelvic CT with oral and intravenous contrast revealed severe mucosal thickening of a short segment of the descending colon measuring 5 cm in length, causing severe lumen narrowing and partial obstruction. No evidence of lymphadenopathy was found on the imaging (Figures 1,2). An infectious workup was performed with blood cultures and stool studies, including *Clostridium difficile* toxin, *Shigella*, *Escherichia coli* O157 H7, ova, and parasites, which were negative. The stool osmolar gap was 64 mOsm/kg excluding secretory and malabsorptive causes. Gastroenterology was consulted, and a colonoscopy was performed, which showed a 4-cm protruding mass with compression noticed in the descending colon, with partial obstruction of the lumen corresponding to the area of CT findings (Figure 3). The area of compression and random biopsies from the entire colon were obtained during colonoscopy and sent for histopathological examination. However, the concern was extraintestinal mass causing a pressure effect, so the area was not tattooed during the colonoscopy. The biopsy from the mucosa of the descending colon showed acute inflammatory changes without crypt abscess, granulomata, parasites, infectious changes, and malignancy. CA19-9 and CEA were unremarkable. The concern was the mass arising from the extraintestinal region causing a pressure effect. After reviewing the CT images with the radiologist, there was a concern about the possibility of malignancy arising from the pelvic adnexa (ovarian malignancy) vs a retroperitoneal benign cyst or malignant tumor with necrosis. USG was performed to evaluate the ovaries and pelvic adnexa for possible primary malignancy, which was not visualized well on the CT scan. Pelvic ultrasound showed a simple ovarian cyst.

**Figure 4.**
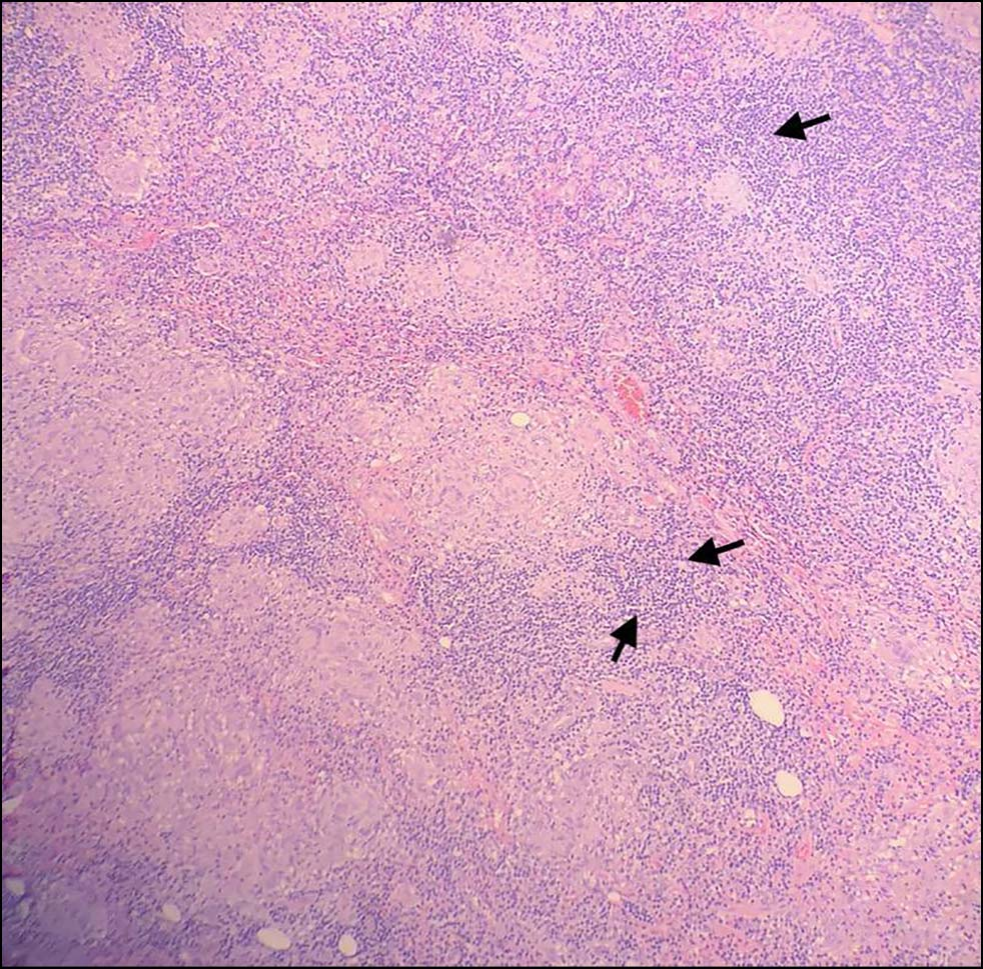
Histopathological results of the left colon segmental resection showing acute inflammation (arrows) and abscess in the pericolic tissue. No firm evidence of inflammatory bowel disease was found in the examined material (H&E 100×). H&E, hematoxylin and eosin.

**Figure 5.**
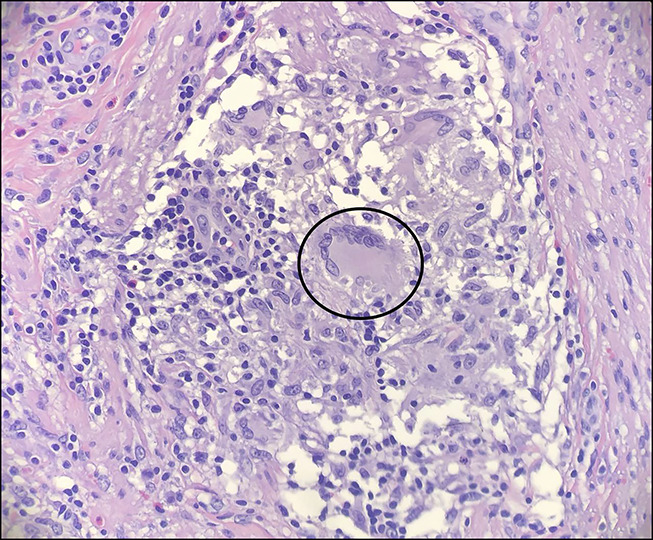
Histopathological results of the appendix showing granulomata (circle) with the involvement of the mucosa, submucosa, muscularis propria, and serosa indicating subacute to chronic appendicitis (H&E 400×). H&E, hematoxylin and eosin.

General surgery was consulted for diagnostic laparoscopy because of concern for retroperitoneal malignancy. A significant inflammatory process was noted in the left lower and right lower quadrants with bilateral omental attachment to the abdominal wall during the diagnostic laparoscopy. On the right side, after the omentum was removed, appendix was noted to be completely stuck to the pelvic wall, which was removed subsequently. On the left side, after removing the omentum, a large abscess cavity was seen on the right gutter between the distal portion of the left colon and the abdominal wall, with penetration of the abscess into the abdominal wall extending in the pelvic region, indicating complex abscess. There was a significant inflammatory process of the distal portion of the left colon with unclear etiology, and the decision was made to remove the distal portion of the colon for the same reason. The histopathology of the left colon segmental resection showed acute inflammation, ulceration, edema, and fibrosis associated with perforation and abscess in the pericolic tissue. 0.3–0.6-cm reactive lymph nodes were noted. The histopathological results of the appendix showed granulomata with the involvement of the mucosa, submucosa, muscularis propria, and serosa. No firm evidence of inflammatory bowel disease was found in the examined material. Acid-Fast Bacilli stain and fungal stain were negative, indicating subacute to chronic appendicitis. No parasites or viruses were noted (Figures 4,5). The postoperative course was unremarkable, and the patient was discharged home with a recommendation to follow up with general surgery.

## DISCUSSION

In clinical practice, chronic or subacute appendicitis is more uncommon than acute appendicitis.^1^ An appendicular abscess occurs between 2% and 10% of patients with appendicitis.^2^ The presentation includes constant abdominal pain, fever, and histologic findings of chronic inflammation.^1^

Only 1 case of chronic appendicular abscess presenting as bowel obstruction and 3 cases mimicking neoplasm have been reported in the literature.^3–6^ Malignant diseases of the cecum causing appendicitis and appendicular abscess mimicking cecal malignancy are common to the proximity. However, appendicular abscess causing descending colon obstruction is quite rare. Our patient's abscess simulated a neoplastic obstructive process of the left descending colon, an unusual presentation, and location. Given the rarity of the presentation, clinicians should consider the unique nature of intra-abdominal abscess secondary to subacute appendicitis that can mimic neoplastic, inflammatory, and infectious processes.

Although acute appendicitis is the most common, patients can also present with nonacute forms such as a subacute or chronic appendicular abscess. The presentation can masquerade as a mass causing bowel obstruction, resulting in an incorrect diagnosis. With the delay in diagnosis, this rare clinical entity poses a diagnostic and therapeutic quandary for clinicians.

## DISCLOSURES

Author contributions: Y. Katamreddy wrote the manuscript and is the article guarantor. T.J. Konturek provided and interpreted the images. O. Fadeyi edited the manuscript.

Financial disclosure: None to report.

Informed consent was obtained for this case report.
